# Filling the gaps: A community case study in using an interprofessional approach and community-academic partnerships to address COVID-19-related inequities

**DOI:** 10.3389/fpubh.2023.1208895

**Published:** 2023-07-20

**Authors:** Marisa L. Kutchma, Julianna Perez, Elizabeth Stranges, Kellie Steele, Tayler Garis, Anastazia Prost, Sumbul Siddiqui, Candice Choo-Kang, Bonnie Shaul, Dede Golda Gbikpi Benissan, Gwendylon Smith-Haney, Nallely Mora, Maya Watson, Thao Griffith, Nathaniel Booker, Amanda Harrington, L. Kate Mitchell, Amy Blair, Amy Luke, Abigail Silva

**Affiliations:** ^1^Parkinson School of Public Health and Health Sciences, Loyola University Chicago, Maywood, IL, United States; ^2^Stritch School of Medicine, Loyola University Chicago, Maywood, IL, United States; ^3^School of Social Work, Loyola University Chicago, Chicago, IL, United States; ^4^Health Justice Project, Loyola University Chicago, Chicago, IL, United States; ^5^Loyola University Medical Center, Maywood, IL, United States; ^6^Internal Medicine, Chinle Comprehensive Health Center, Indian Health Service, Chinle, AZ, United States; ^7^AbbVie, Chicago, IL, United States; ^8^School of Law, Loyola University Chicago, Chicago, IL, United States; ^9^Law School, Wayne State University, Detroit, MI, United States; ^10^Loyola University Health System, Loyola University Chicago, Department of Pathology and Laboratory Medicine, Maywood, IL, United States; ^11^Village of Maywood, Maywood, IL, United States

**Keywords:** interprofessional, community-academic partnership, COVID-19, health equity, interdisciplinary collaboration, public health entrepreneurship

## Abstract

Public health challenges rapidly escalated during the COVID-19 pandemic. In response to a severe lack of resources and support in the near western suburbs of Chicago, the COVID Equity Response Collaborative: Loyola (CERCL) was established by an interprofessional team of Loyola University Chicago students, staff, and faculty. CERCL sought to minimize the negative impact of COVID-19 on vulnerable communities, those that are largely Black, Hispanic, or low-income. From April 2020 to the present, the collaborative utilized community-academic partnerships and interdisciplinary collaborations to conduct programming. CERCL’s programming included free community-based testing, screening for and assistance with social determinants of health, dissemination of relevant and reliable COVID-related information, provision of personal protective equipment, and facilitation of access to vaccines. With partners, the collaborative conducted 1,500 COVID-19 tests, trained 80 individuals in contact tracing, provided over 100 individuals with specifically tailored resources to address social and legal needs, distributed 5,000 resource bags, held 20 community conversations, canvassed 3,735 homes, and hosted 19 vaccine clinics. Community-academic partnerships with the health system, community and governmental agencies, and the local public health department have been critical to CERCL efforts. The interdisciplinary and interprofessional successes demonstrated in this case study lends the example of a relevant, sustainable, and practical intervention to address nuanced public health issues.

## 1. Introduction

By February 2020, it was apparent that COVID-19 was a public health emergency. In addition to a lack of preparedness and collaboration within medical and public health systems, issues such as systemic racism, decreased public trust, and chronic community and public health disinvestment created a breeding ground for the crisis which followed (1–4). Since the 2008 recession, the US public health system has faced notable reductions. The local health department (LHD) workforce capacity has been reduced by 21% with larger LHDs being affected to a greater degree (5). Spending has been reduced by 16% *per capita* for state health departments and 18% *per capita* for LHDs (6). Nationwide, the number of full-time equivalent employees in LHDs decreased ~16% from 162,200 in 2008 to 136,000 in 2019; full-time equivalent employees in state health departments decreased ~12% from 92,000 in 2008 to 81,000 in 2019 (6). Some LHDs were better resourced to respond to the needs of their communities while underfunded LHDs, many in under-resourced areas, left vulnerable communities to fend for themselves. The Loyola University Chicago (LUC) Health Sciences Campus is located in the Village of Maywood, a low-income medically underserved community in the near western suburbs of Chicago (7). The community’s LHD is the Cook County Department of Public Health (CCDPH), which serves suburbs surrounding the city of Chicago. The city is, respectively, served by the Chicago Department of Public Health. The city of Chicago is home to 2.7 million people (8) and allocated $55 million to the Chicago Department of Public Health in 2020 (9). The Cook County Department of Public Health serves approximately an equal-sized population (2.5 million people), spread across 125 racially and economically diverse municipalities, however, CCDPH had a considerably lower budget of $10.2 million (10–11). This insufficient funding and staffing infrastructure left the system vulnerable to being overwhelmed and unprepared for COVID-19.

COVID-19 has disproportionately impacted Hispanic and Black communities (12–14). The LUC Health Sciences Campus and three affiliated hospitals are located among some of the hardest-hit communities in West Suburban Cook County (WSCC) (15). Five of these communities: Bellwood, Berwyn, Cicero, Maywood, and Melrose Park, are within the service area of the affiliated hospitals. In July 2020, these communities accounted for 17.1% of cases and 9.3% of COVID-related deaths, despite the combined populations representing only 8.0% of the population in CCDPH’s coverage area (15–18). These five communities are distinctly communities of color: Berwyn, Cicero, and Melrose Park residents mostly identify as Hispanic (63.2, 89.2 and 74.2%, respectively), while Bellwood and Maywood are predominantly Black (77.3 and 68.3%, respectively) but with growing Hispanic populations (17.9 and 27.3%) (18). These communities are impacted by structural racism (i.e., segregation, “white flight,” poverty, and unequal health care access) which increased their vulnerability to COVID-19 infection and subsequent negative outcomes (2, 19–23).

## 2. Context

Early in the pandemic, Maywood community leaders alerted LUC Health Sciences Campus faculty about the lack of free and accessible testing. At the time, neither the medical center nor any other private or public entity was addressing the disproportionately high case rate in Maywood. This call cultivated a spirit of public health entrepreneurship through which faculty, staff, and student volunteers in the sectors of public health, medicine, health care administration, nursing, law, and social work rapidly came together to address the unmet need (24). In April 2020, the COVID Equity Response Collaborative: Loyola (CERCL) coalesced and sought to use an interprofessional approach and forge community-academic partnerships to mitigate the harmful impacts of COVID-19 on the most vulnerable populations in WSCC.

Both interprofessional collaboration and community-academic partnerships are essential in public health emergencies (25). Interprofessional teams can quickly pivot to address evolving needs and provide comprehensive support for communities (26). Community-academic partnerships are developed around values and themes that include community involvement, collaboration, communication, best practices, relationship building, clarity, and strategic planning (25). These were central to CERCL’s framework as we partnered with academic, health system, community, public, and governmental allies, and teamed up with qualified medical volunteers (e.g., physicians, nurses, faculty, and students) to address the gap in freely available COVID-19 testing in underserved WSCC communities. As community needs shifted, so did CERCL’s approaches. This article illustrates the various strategies used by CERCL to support equity in access to testing, vaccines, and other resources to abate the disproportionate harm of the pandemic on Hispanic and Black communities.

## 3. Programmatic elements

CERCL’s strategy was informed by the ‘Box-it-in’ approach, which consists of testing, isolating those with infection, identifying contacts of those infected, and quarantining contacts (27). To that end, CERCL sought to: (1) deliver and expand free testing in the community; (2) build contact tracing capacity through the provision of free training; and (3) aid communities by providing information on local and freely available resources to support quarantining, isolating, and safety.

To obtain insight on best practices, CERCL looked to community-based organizations and clinics that were conducting pop-up and drive-through testing to understand the logistics and resources needed. An intramural grant awarded in the summer of 2020 funded COVID-19 testing as well as a contact tracing training program.

### 3.1. Organization

The following seven interprofessional teams were developed to accomplish the work and led by a faculty or clinical team member: Volunteer Coordination, Data Collection, Logistics, Test Results, Resources, Publicity, and Contact Tracing (Table 1). CERCL team meetings were held throughout the week including the leadership meeting which was open to all members. The leadership meetings were used to guide the overall direction and mission of the community-academic partnership and hold space to report achievements and next steps.

**Table 1:** Description of the COVID equity response collaborative’s interprofessional teams and community-academic partnerships.

Interprofessional teams	Composition	Purpose	Outcomes
Volunteer Coordination	*Medicine and nursing faculty, medicine and health care administration students*	Recruit and train bilingual student volunteers to carry out the various aspects of the testing (e.g., intake personnel, testing assistant) and for returning test results.	A total of 99 student volunteers and 57 medical volunteers were recruited and trained.
Data Collection	*Public health and nursing faculty*	Identify the data elements needed for testing and subsequently research. Develop the databases needed to help automate the work.	Created a REDCap project capable of gathering and managing data, collecting electronic signatures for consents, and emailing test results.
Logistics	*Medicine and nursing faculty*	Develop protocols for safe testing. Ensure testing sites have all needed supplies.	Created site-specific protocols for testing and developed an inventory tracking system.
Test Results	*Medicine faculty and students*	Create scripts for delivering test results. Provide test results utilizing these scripts.	Provided results to all those who were tested at community sites.
Resources	*Law faculty, law and social work students, AmeriCorps VISTAs*	Develop a screening tool to identify health-harming needs and create resource sheets that highlight local and either free or sliding-scale services.	Followed up with any client that requested to be contacted (*via* the screening tool) for services, advice, or guidance.
Publicity	*Public health, medicine, healthcare administration, and law students*	Create and disseminate bilingual (Spanish and English) materials (e.g., banners, flyers, website) to advertise testing and promote relevant COVID-19 information.	Helped design a website and produce its content, created a social media presence, and physically disseminated information (e.g., posted flyers, handed out flyers at events) in the community.
Contact Tracing	*Public health faculty and students*	Develop and deliver a freely available training focused on soft skills to help the public health department build its contact tracing capacity.	Created a set of 6 on-demand online modules and a hands-on workshop that was freely available to students and community members.

Community-academic partnerships	*Institutions*	Role	Outcomes
Coalition for Spiritual & Public Leadership	*Casa Esperanza, Rock of Ages Baptist Church*	Allowed the use of their open-air parking lots for CERCL to deliver safe and free drive-thru or walk-up testing. Facilitated relationship building with community partners and fostered community involvement.	Free testing available to the communities (Melrose Park and Maywood) twice a week (July–September 2020).
City of Berwyn	*Berwyn Public Health District*	Donated space in their clinical offices for CERCL to conduct free testing.	Free testing available to the community once a week (January–April 2021).
Village of Maywood	*Mayor’s Office, Community members*	Provided various spaces and opportunities for CERCL to conduct free testing and host vaccination clinics. Participated in CERCL’s strategic planning and provided clarity to programming. Promoted communication with community by sharing CERCL resources, events, and services. Fostered relationship building by tying in additional partners. Community members volunteered in outreach and information sharing.	Free testing available to the community at least once a week (November 2020 – October 2022). Ongoing vaccination clinics at community events.
Village of Bellwood	*Mayor’s Office*	Donated space in their youth center for CERCL to conduct free testing.	Free testing available to the community once a week (March–July 2021).
State of Illinois	*Public Health Department*	Provided free vaccinations and staff support to deliver them. Contributed to evidence-based best practices for vaccination clinics.	Co-hosted more than 19 vaccination clinics between April 2021 and February 2022.
Cook County in Illinois	*Public Health Department*	Provided free vaccinations and staff support to deliver them. Also provided informational material and face masks. Contributed to evidence-based best practices for vaccination clinics. Provided speakers for some community conversations events.	Co-hosted 5 vaccination clinics between April 2022 and present. Donated 1,000 K95 masks as well as bilingual COVID-19 information materials.
Village Free Press	*Local newspaper*	Helped promote free testing and vaccination events and supported COVID-19 information dissemination activities. Co-hosted community conversations.	Published advertisements on their online and print versions of their weekly paper and hosted various virtual bidirectional community conversations on topics related to COVID-19.
Loyola University Health System	*Clinical Microbiology Laboratory, Pediatric Mobile Health Unit, Student and faculty volunteers*	Subsidized COVID-19 test kit and laboratory testing; provided free pediatric services (e.g., immunizations, physical exams, health education, and screenings; e.g., lead, asthma, obesity). Contributed to upholding best practices for COVID-19 testing. Loyola students created flyers and worked on CERCL’s social media.	Supported all free testing available in the communities (November 2020–October 2022). Provided pediatric services to more than 100 children (July 2020-present).
Garden House of Maywood Apartments	*Social Coordinator*	Co-hosted vaccination and outreach events. Provided space to host events.	Co-hosted 2 vaccination events in 2022 for seniors in the community.
Maywood Park District	*Lightford Recreation Center*	Provided space to host testing and vaccination events. Participated in communications by sharing CERCL information, events, and resources.	Free testing available to the community (November 2020 – October 2022). Involved in 8 vaccination events.
AmeriCorps	*VISTA*	Coordinated CERCL’s relationship with the LUC School of Law’s Health Justice Project. This role was central to strategic planning and relationship building.	Distributed resource information and updates through social media, CERCL’s website, and at community events (August 2021-present).

The Volunteer Coordination team responded to staffing needs surrounding COVID-19 testing. This team ensured that all events were adequately staffed and volunteers were fully trained and informed.

The Data Collection team created a system for uniformly collecting data through a single web-based secure platform developed for this project. It was used to capture basic demographic data, track test results, and collect survey data (for those who consented to research).

The Logistics team created and implemented safety protocols at the testing sites. They also managed the inventory and ensured that the testing sites were stocked with all the necessary materials.

The Test Results team was responsible for maintaining phone call and email scripts to be used for delivering test results. These scripts included vital and up-to-date information from the Centers for Disease Control and Prevention.

The Resources team collated information on local and freely available social and legal resources which were shared with testing participants. Needs were identified through a brief screening during intake. Team members then followed up with individuals who requested further assistance.

The Publicity team was charged with creating CERCL’s website as well as content to canvas and inform communities about the collaborative’s COVID-19 testing efforts. Alongside this, the team worked to develop frequent social media coverage regarding CERCL and community resources on the platforms of Facebook, Instagram, Twitter, and YouTube. The team facilitated the collaborative’s relationship with the local newspaper.

The Contact Tracing team worked to increase the number of qualified contact tracers in the area by building an on-demand online module as well as offering periodic workshops to practice soft skills. These programs were freely available to university students and community members.

### 3.2. Establishing community partners

CERCL’s Community Liaison is an individual who holds a strong connection to Maywood and community engagement. Their role helped ensure culturally informed practices as well as facilitated outreach in the Village. They acted as a conduit connecting community and academic partners (Table 1). CERCL volunteers reached out to community leaders with whom the Community Liason and other CERCL team members had existing relationships in order to identify regular testing locations. Two community partners were immediately identified through the Coalition for Spiritual and Public Leadership: a place of worship in Maywood and a community center in neighboring Melrose Park (Table 1).

Several trusted governmental allies in Maywood, Bellwood, and Berwyn approached CERCL regarding partnership for free testing and provided spaces in their communities. Not only did the local leadership co-host events, but they also provided resources to do so. Maywood leadership was central to decision making in carrying out vaccination, testing, and outreach campaigns. Additionally, both the state and county public health departments facilitated vaccination efforts by co-hosting clinics in community locations or events. The local newspaper also became a consistent partner, helping to raise awareness about testing and vaccine availability as well as disseminating COVID-related information.

The Loyola University Health System provided nurses to assist with testing, free nasopharyngeal swab RT-PCR testing kits, and processed samples at no cost. These contributions greatly reduced the overall financial burden of testing. CERCL partnered with the Loyola Pediatric Mobile Health Unit to further expand COVID-19-related resources by providing free general pediatric services on a monthly basis at the testing sites or other community locations. Among the services offered were physical exams, immunizations, health education, and lead screening.

AmeriCorps VISTA, a national service program that addresses poverty and strengthening of communities, provided volunteers through the Health Justice Project (a medical-legal partnership with LUC’s School of Law) to support CERCL by identifying and disseminating information on resources, screening participants for social and legal needs, and linking participants to resources as needed.

### 3.3. Funding and sustainability

The CERCL team applied for continued funding to expand testing and resources. In the fall of 2020, CERCL successfully obtained additional funding from the county public health department to disseminate personal protective equipment (PPE) as well as to execute a local, bilingual, and multi-channel outreach and education campaign delivering information to the community regarding testing, contact tracing, quarantine and isolation, and legal rights. In addition, this funding provided an opportunity to develop and disseminate materials regarding health care, housing, food, and legal resources freely available in the community.

Also in fall 2020, CERCL received funding from a local foundation to expand testing to additional locations and provide community residents an opportunity to participate in COVID-19 research. The CERCL team identified research as a priority to contribute to preparing for future pandemics. The research consisted of: (1) using a questionnaire to identify possible transmission routes through social network and epidemiologic analysis; (2) biobanking nasopharyngeal and serum samples for emerging pathogen surveillance; and (3) conducting serologic surveillance of immune response through serial antibody testing. Consent and participation of the community members in the research were completely voluntary and did not affect their ability to engage in CERCL initiatives. The research was considered an additional service offered to the community.

## 4. Outcomes

### 4.1. Free community-based testing

Between July 2020 and October 2022, community COVID-19 testing took place in 4 communities of WSCC: Maywood, Melrose Park, Bellwood, and Berwyn (Table 2). Testing was completely free and did not require identification or insurance information. The locations included: a parking lot, recreation center, clinical office, and youth center. More than 1,500 free COVID-19 tests were conducted. The duration of services at each location varied depending on testing attendance and staff availability over time. As the pandemic and available resources changed, CERCL congruently evolved. The team continued identifying community partners and allies to expand free testing in the area as needed. In December of 2021, prior to the surge of the Omicron Variant, the COVID-19 case rate in Maywood was 14,325 cases per 100,000 population while Suburban Cook County’s case rate was 13,897. Case rates were higher in Bellwood (16,770), Berwyn (16,140), and Melrose Park (18,269) (15).

**Table 2:** Description and outcomes of the COVID equity response collaborative’s adapted box-it-in approach to the pandemic.

Approach	Impact
Deliver and expand testing	Provided free and accessible COVID-19 testing in varying locations of West suburban Cook County where access was limited, and the case rates were high. CERCL has provided more than 1,500 tests to community residents in or near five towns and villages.
Build contact-tracing capacity	Created a freely accessible program that included soft-skills training to improve communication and optimize effectiveness of the outreach in diverse communities. A total of over 80 students and community members completed the online on-demand training and over 40 participated in the interactive workshops.
Provide support to communities: *resource information*	More than 1,100 testing participants were screened for social and legal needs. For those that requested a follow-up for their needs, 455 calls were made to provide information on specific resources. In total, ~110 received specifically tailored resource information, and additional individuals received this tailored support.
Provide support to communities*: PPE/resource bags*	More than 5,000 personal protective equipment (PPE)/Resource bags were distributed in the community. These contained face masks, sanitation wipes, a magnet with CERCL contact information, general community resource sheets, and COVID-19 FAQs.
Provide support to communities: *information dissemination through community conversations*	Implemented 20 virtual, live-streamed, interactive (English and Spanish) sessions on COVID-19-related topics in partnership with experts and a local media outlet. More than 3,500 viewers were reached.
Provide support to communities: *information dissemination through* a *website and social media*	Shared information through a robust and up-to-date website and the use of Facebook, Instagram, Twitter, and YouTube to further engage with the community. Created and disseminated two public service announcements.
Provide support to communities: *information dissemination through community canvassing*	10 trained vaccine ambassadors went door-to-door to conduct relevant and sensitive conversations regarding COVID-19 and vaccinations while distributing PPE/resource bags. They canvassed 3,735 homes with 941 answering the door and engaging in conversation.
Provide support to communities: *vaccination events*	Co-hosted 19 vaccine clinics at various community events. More than 630 vaccines were distributed and included the first dose, second dose, and all approved boosters for children and adults.

### 4.2. Contact tracing training

More than 80 students and community members completed six online on-demand training modules and approximately 40 participated in the interactive workshops. The six online training modules consisted of topics including the Social Determinants of Health, Cultural Humility, Stages of Change, Problem Solving, Motivational Interviewing, and Professionalism. A job board site and listserv were also created to keep participants informed of contact tracing opportunities.

### 4.3. Supporting communities

At testing sites, over 1,100 participants were screened for social and legal needs and offered information on local and freely available resources. From July 2020 through December 2021, 1,038 individuals were screened using the I-HELP questionnaire, which evaluates health-harming needs. The most frequently reported unmet needs included financial struggles (50%) and loss of income (50%). In addition, no health insurance (25%), remote education access concerns (25%), lack of primary care provider (24%), safety in home (12%), and legal/court dates (8%) were cited as well. In total, 434 individuals (~42%) requested follow-up after taking the questionnaire. Of those, almost 27% were successfully connected with resources pertaining to food, health insurance or health care, utilities, legal advice, PPE, and more.

In addition, more than 5,000 bags with PPE (N95 masks, KN95 masks, and surgical masks) and resource information (in English and Spanish) were distributed during canvassing and a variety of community events. The contact information included in these bags allowed community members and their networks to stay in touch with CERCL through our Google Voice line. To date, the line has received over 700 voicemails in addition to calls answered and placed.

Community members also received COVID-19-related information through a series of 20 online interactive community conversations. Topics included COVID-19 therapeutics, the COVID-19 vaccine, COVID-19 myths and facts, how to stay healthy during COVID-19, and more. Community conversations were held every few months on assorted topics that were deemed to be of critical and timely importance based on community and staff feedback. The Village Free Press partnered with CERCL in many of these events and provided accessible live platforms through Facebook and YouTube.

CERCL continued its outreach through its website, social media platforms, and available printed material at the testing sites and community events. The website and social media content were regularly updated and expanded to relay relevant and reliable information to community members.

### 4.4. Pivoting to vaccinations

CERCL employed strategies to facilitate access to vaccinations once they became available. The strategies included neighborhood canvassing, assistance with vaccination appointments, and co-hosting vaccination clinics with partners (state and county public health departments, community organizations, and governmental agencies).

As the demand for testing decreased and the need for vaccinations increased, CERCL shifted the collaborative’s approach to outreach. Neighborhood canvassing was implemented in the summer of 2021. This involved graduate students who were trained as vaccine ambassadors according to the Malcolm X College Trusted Vaccine Ambassador Program Course (28). This training highlighted key information surrounding COVID-19, the vaccine, and respectful conversations centering cultural humility. Vaccine Ambassadors canvassed door-to-door in the Maywood community, providing bags that contained PPE, CERCL and community resources, and a refrigerator magnet with CERCL’s contact information. During canvassing, volunteers collected information on vaccine uptake and hesitancy.

About 941 (25%) of the 3,735 Maywood homes that were canvassed had residents answer the door. Of those who answered the door, 669 homes had residents who were vaccinated (1,729 vaccinated individuals total). There were 138 homes with residents who were unvaccinated citing various reasons. The most commonly endorsed reasons for being unvaccinated included general disinterest, upcoming plans to obtain vaccination, and side effects or medication interactions. This project cultivated open conversations and transparent, bidirectional information sharing.

Furthermore, CERCL co-hosted over 19 vaccination events resulting in more than 630 vaccinations. As the pandemic evolved, clinics continually shifted to incorporate official guidelines, which included offering booster shots and pediatric doses. The clinics in which the greatest numbers of vaccinations were distributed were those in partnership with other community organizations. These tended to reach a greater number of folks than stand-alone clinics. In May 2022, vaccination rates in Maywood, Suburban Cook County, and the US were 57.2, 66.0, and 66.3%, respectively (29, 30).

### 4.5. Community-academic partnerships

The community-academic partnership model was critical to achieving positive outcomes in CERCL. Teamwork, network sharing, and event collaboration allowed CERCL and coalition partners to reach more community members in efforts to promote health equity and build trust. These partnerships shaped how CERCL services were implemented and improved. Community members and partners developed the walk-up testing model, selected the most accessible locations for services and resource sharing, and provided input on the most helpful aspects of programming as well as areas needing improvement. Weekly interdisciplinary CERCL meetings continue to be held.

## 5. Lessons learned

As a result of CERCL’s work since the onset of the pandemic, the team has learned from partners and community members, as well as programming successes and areas for needed improvement. These invaluable lessons have shaped CERCL’s services and outreach and can be useful to anyone looking to enact change at the local level. Leveraging community and academic entities in this community case study allowed CERCL to engage in meaningful, practical, and relevant work in WSCC.

### 5.1. Teamwork: maintain a nimble approach to complexity

Pivoting the focus of large, complex public health and health care systems toward community needs proved difficult in the early, emergency phase of the pandemic. The focus on treating known, severe COVID-19 cases through building intensive care unit and hospital bed capacity was essential but deprioritized upstream public health interventions such as the ‘Box-it-in’ method. When public health departments utilized these same health care systems to deploy testing or vaccination, these assumed health care systems were the trusted community voice and had knowledge of critical social care supports.

CERCL leaders identified that an intermediary was necessary to bridge the gap between complex health systems and community residents. It was also clear that this intermediary needed to be aware of community experiences and center them amid the changing health care system and public health department guidance. Throughout the evolution of national public health recommendations on testing and the development of vaccine campaigns, CERCL delivered community-responsive services to Black and Hispanic communities disproportionately impacted by the pandemic.

### 5.2. Harness the skills of interprofessional teams

Interprofessional collaboration requires *relationships*. Community-academic partnerships were developed in Maywood, Melrose Park, Bellwood, and Berwyn, and were operationalized through CERCL team members and pivotal stakeholders. The Community Liaison role was particularly involved in this process. Once a connection was made, a point person from CERCL would typically act as the conduit between the partners to facilitate event planning, resource sharing, and meetings. At our academic center, we built relationships between the Schools of Public Health, Medicine, Nursing, Law, and Social Work. Interprofessional practice requires *action*. The pandemic unlocked our academic approach and turned education into practice. This coincided with the ubiquitous sense of helplessness among students, staff, and faculty in the early days of lockdown. Many with expertise in community-engaged public health and law were asking ‘How can we help the community safely?’. Our team harnessed skills in fields such as epidemiology, infection prevention, and health care administration by finding and honing methods to safely embed support and resources in the community. With a team of faculty and students, we were also able to harness intergenerational skills to reach both younger and older demographics through social media and in-person community efforts. Furthermore, partner input was sought at all stages in programming and was vital to the successful planning of events and distribution of resources. However, CERCL is intent on incorporating more community input into future program planning and implementation to further build trust and partnership. This will continue to be prioritized as strategic planning is currently being carried out in conjunction with the Village of Maywood Mayor.

The spirit of public health entrepreneurship cultivated interprofessional teamwork surrounding the community-academic partnership, fostering the implementation of design thinking, resource mobilization, financial viability, cross-disciplinary collaboration, and systems strengthening included in the framework imparted by Chahine (31). Design thinking was implemented in the development of CERCL programming on-the-go. Testing and vaccination models were implemented and adjusted accordingly with gaining understanding at each event and iteration. The collaborative mobilized resources by gradually obtaining more funding and support, while utilizing limited resources to accomplish a wide range of activities. The financial viability of CERCL has centered around minimizing inputs and is currently being cultivated as the team is trying to shift to a non-profit approach. Cross-disciplinary collaboration is deeply ingrained in the collaborative’s mission as it is a foundational value of the team. Systems strengthening has been key to CERCL’s vaccination efforts as CERCL has taken a role in larger campaigns enacted at state and county levels (31).

### 5.3. Focus on the most vulnerable

In the early stages of the pandemic and the early months of vaccination, those with the greatest number of resources were the most likely to get tested and receive vaccines (2, 32, 33). By collaborating with the communities that struggled with testing access, vaccine acceptance, and overall distrust, we were focusing on the area of greatest impact and the communities most likely to get left behind. Existing structural factors increased the risk of transmission and mortality for Black and Hispanic communities because of discrimination, lack of health care access and insurance, education, income, and wealth gaps. The communities in CERCL’s service area also had higher numbers of essential workers than surrounding suburbs with higher average incomes, making them even more vulnerable to COVID-19 exposure (34). These communities were unable to shelter in place, work from home, or readily get online to sign up for the limited vaccination appointments as soon as they would be released. CERCL focused on those who did not have the resources, time, or capacity to incorporate pandemic challenges into their daily lives by creating a critical network of social and legal resources and connecting Black and Hispanic communities.

### 5.4. Identify trusted community voices

Prior to the pandemic, we observed that the community had lost trust in local health systems due to changing dynamics in ownership and barriers to accessing care. A health care system that makes communities feel abandoned will not be a trusted resource (35, 36). Trust in science, health care systems, and each other is key to the collective action that unified some other nations during the earlier stages of the pandemic (37). When distrust of or abandonment by larger systems is the reality, local solutions are needed. We greatly depended on community liaisons and a network of community leaders to build bridges, normalize the need for testing, answer questions about vaccination, provide related social and legal resources, and continue to show accompaniment during uncertainty. Community stakeholders played an important role in fostering pre-existing relationships with the community and strengthening CERCL’s community ties. This collective action filled the gaps left by the health system and forged new collaborations. CERCL would not have accomplished the same programming without these relationships and trust. Trusted community voices are those that are also the best listeners, who listen to understand without agenda, maintain respect, and acknowledge the historical and political root causes of disinvestment.

## 6. Conclusion

CERCL has shifted our model of delivery as the pandemic and available resources have evolved. In order to continue working to best meet the needs of the most vulnerable communities in WSCC, CERCL is transitioning to a more general responsive community health equity collaborative model. The ‘C’ in CERCL will stand for ‘Community’ rather than ‘COVID’. The Community Equity Response Collaborative: Loyola will continue to advocate through interprofessional collaboration and pivot to respond to the present needs and strengths of the community-academic partnership. CERCL continues to find novel approaches to promoting community-academic partnership in meaningful ways as well as supporting trust and ultimately health equity.

Currently, the collaborative is continuing to engage in vaccination and information dissemination efforts in WSCC. A community engagement fellowship has also been established, which links qualified students and local organizations to further develop community-academic partnerships. The number of partnerships is growing and involve addressing broader community needs, including gun violence prevention and mental health.

A recent strategic planning session was also held to coordinate future programming for CERCL as the public health environment in Maywood and Cook County changes. Along with this, a community assessment is being planned to further promote the sustainability of CERCL and to gain a deeper understanding of the changing strengths, weaknesses, opportunities, and threats in WSCC by listening to those who live, work, and interact in the area. This will inform more feasible and effective programming in this post-peak of COVID-19 pandemic era. As the COVID-19 pandemic and the overall environment of public health evolve, we look to further enhance our public health entrepreneurship methodology, advocacy efforts, and community networking.

## Data availability statement

The original contributions presented in the study are included in the article/Supplementary material, and further inquiries can be directed to the corresponding author.

## Author contributions

MK, JP, GS-H, AL, and AS contributed to conception and design of the study. MK and JP wrote the first draft of the manuscript. AB and AS wrote sections of the manuscript. All authors contributed to manuscript revision, read, and approved the submitted version.

## Funding

This work was supported by the Walder Foundation’s Chicago Coronavirus Assessment Network (Chicago CAN) initiative, Suburban Cook County COVID-19 Contact Tracing Community Supports initiative, and the Loyola University Health EQ Collaborative.

## Conflict of interest

The authors declare that the research was conducted in the absence of any commercial or financial relationships that could be construed as a potential conflict of interest.

## Publisher’s note

All claims expressed in this article are solely those of the authors and do not necessarily represent those of their affiliated organizations, or those of the publisher, the editors and the reviewers. Any product that may be evaluated in this article, or claim that may be made by its manufacturer, is not guaranteed or endorsed by the publisher.
